# A CNN-Based Wearable System for Driver Drowsiness Detection

**DOI:** 10.3390/s23073475

**Published:** 2023-03-26

**Authors:** Yongkai Li, Shuai Zhang, Gancheng Zhu, Zehao Huang, Rong Wang, Xiaoting Duan, Zhiguo Wang

**Affiliations:** Center for Psychological Sciences, Zhejiang University, Hangzhou 310028, Chinazhiguo@zju.edu.cn (Z.W.)

**Keywords:** drowsiness detection, glass, lightweight, driving, convolution neural network

## Abstract

Drowsiness poses a serious challenge to road safety and various in-cabin sensing technologies have been experimented with to monitor driver alertness. Cameras offer a convenient means for contactless sensing, but they may violate user privacy and require complex algorithms to accommodate user (e.g., sunglasses) and environmental (e.g., lighting conditions) constraints. This paper presents a lightweight convolution neural network that measures eye closure based on eye images captured by a wearable glass prototype, which features a hot mirror-based design that allows the camera to be installed on the glass temples. The experimental results showed that the wearable glass prototype, with the neural network in its core, was highly effective in detecting eye blinks. The blink rate derived from the glass output was highly consistent with an industrial gold standard EyeLink eye-tracker. As eye blink characteristics are sensitive measures of driver drowsiness, the glass prototype and the lightweight neural network presented in this paper would provide a computationally efficient yet viable solution for real-world applications.

## 1. Introduction

Drowsiness is a “state of low alertness” [1], i.e., “a tired state, between sleeping and being awake” [2]. Previous researches have shown that drowsiness is a leading cause of traffic accidents that result in serious injury or death [3,4,5]. According to a report by the AAA Foundation for Traffic Safety, from years 2009 to 2013, 21% of fatal crashes involved a drowsy driver [6]. In a recent naturalistic study, more than 3500 drivers were monitored for months with in-vehicle cameras and sensors while they drove their cars. The results showed that drowsiness, quantified with the percentage of eye closure (PERCLOS), was identified in 10.6–10.8% of the crashes that caused significant property damage or injury [7]. To improve road safety, various in-vehicle sensing technologies have been employed to detect driver drowsiness in real-time [8,9,10]. Most notably, researchers have frequently resorted to camera-based solutions that rely on various visual features of the eye, mouth, head, or body to assess driver states, e.g., fatigue or distraction [11,12,13].

The cameras used to detect drowsiness are typically installed inside the car (e.g., on the dash), providing a remote-sensing solution that has no physical contact with the driver. This remote camera solution is more driver-friendly than wearable sensors such as EEG and ECG; however, the camera is an add-on that is difficult to integrate into the consoles of existing vehicles. While car makers are moving forward to install remote camera-based drowsiness detection devices as part of their advanced driving assistance systems (ADAS), the immediate impact of this solution is limited as it is impractical to install such devices on a sizable portion of the estimated 1.446 billion vehicles already running on the road [14]. Furthermore, drowsiness detection devices that rely on in-cabin remote cameras need to process human face, eye, head, and body features in a complex and fast-changing driving environment where the lighting condition varies from time to time. The algorithm is complex and the computing power required for the onboard computer is high. Last but not the least, camera-based drowsiness detection devices inevitably cause privacy concerns and may hinder adoption among drivers.

Wearable drowsiness detection devices are less ideal when user comfort is concerned; however, wearable devices such as glasses do have several advantages over the in-cabin remote camera solution. Most importantly, the sensors (e.g., camera or proximity sensor) on wearable devices are close to the eyes and thus can easily obtain high-quality data that we can use to extract eye states (e.g., blink) or gaze information. This greatly simplifies the drowsiness detection algorithms. Additionally, the sensors monitor only part of the human face (mostly the eyes) and are less prone to privacy issues. Compared to wearable devices such as EEG and EOG, glasses are more acceptable as many people wear sunglasses for protection against glare while driving.

This paper reports a wearable drowsiness detection system that images the eye area via a hot mirror and uses a lightweight convolution neural network to measure the eye area. The eye area was used to estimate the widely used eye closure measures that are sensitive to drowsiness, i.e., PERCLOS and blink rate. We also compared the output of this wearable device to an industrial gold standard EyeLink eye-tracker. The main contributions of the present work are summarized as follows:

- built a wearable glass that can be used to extract drowsiness metrics such as PERCLOS and blink rate under the challenging lighting conditions of everyday driving;
- created a real-time Lightweight Glass Network (LGN) capable of reliably monitoring eye-opening with a budget camera and affordable wearable computing device;
- benchmarking tests show that the LGN-based wearable glass can detect blinks with an accuracy comparable to industrially recognized EyeLink eye trackers.

The rest of this paper is organized as follows: Section 2 briefly reviews two lines of research work closely linked to the research topic of this paper. Section 3 describes the hardware design of the glass prototype and the architecture of the Lightweight Glass Network (LGN). The experiment used to validate the system output is described in Section 4, and the results of the experiment are presented and discussed in Section 5. Section 6 concludes the paper with a discussion of future research work.

## 2. Related Work

Previous studies have examined three categories of measures for drowsiness detection, i.e., physiological measures (e.g., EEG; for a review, see [15]), vision-based measures (eye closure, yawning, head pose, etc.; for a review, see [8]), and driving dynamics (lane position, speed control, etc.; for a review, see [16]). Driving dynamics can be extracted from in-vehicle or smartphone sensors. However, these measures are an indirect measurement of the driver’s state. Physiological and vision-based measures, on the other hand, provide direct drowsiness measures via wearable (e.g., EEG electrodes) or remote sensors (e.g., eye-tracking cameras). The physiological measures (e.g., alpha wave in EEG) are the most sensitive ones; however, vision-based measures are more promising for large-scale consumer adoption. Related to the present work, both physiological and vision-based methods can be used to measure eye features sensitive to driver drowsiness (e.g., blink rate and PERCLOS). 

### 2.1. EOG-Based Drowsiness Detection

Before video-based eye-tracking was widely available, electrooculography (EOG) was a technology psychologists used to track human eye movements (see [17], for a review). In addition to gaze information, the EOG signal also contains abundant information about the eyelid movements which can be used to derive measures sensitive to driver drowsiness, e.g., blinks or PERCLOS (for a recent review, see [18]). For instance, in a recent study by Xue et al. [19], EOG electrodes were used to extract an eye closure measure, which was then used as a key parameter to assess driver fatigue. Schmidt et al. [20] developed a very detailed algorithm for blink detection in EOG signals and compared EOG-based blink measures with those extracted from eye-tracking cameras in manual and automated driving scenarios. EOG-based eye metrics have proven useful in lab tests; however, such devices are difficult to implement in real-world driving scenarios due to environmental noise and user acceptance.

### 2.2. Camera-Based Drowsiness Detection

Compared with EOG-based eye feature measurement, camera-based devices can be installed inside the cabin to provide a remote sensing solution that requires no physical contact with the driver (see [21] for a recent review). In the past two decades, various algorithms have been developed to retrieve visual features that are sensitive to driver drowsiness. For instance, Maior et al. [22] developed a deeply learned facial expression analysis algorithm that used eye and mouth features to detect driver fatigue. By extracting the eye aspect ratio and mouth aspect ratio from facial landmarks, Cheng et al. [23] built a model to assess fatigue with logistic regression. Bamidele et al. [24] designed a non-intrusive and low-cost driver drowsiness detection solution based on face and eye tracking. Madireddy et al. [25] built a non-intrusive drowsiness detection system based on Raspberry Pi and OpenCV. In their system, an SVM was employed to extract visual features, such as eye and mouth aspect ratios, blink rate, and yawning rate. Kumar et al. [26] utilized a camera and Harr’s feature classifier to extract the eye area for driver drowsiness detection.

Eye and mouth features are the most frequently used features for remote camera-based drowsiness detections [22,23,24,25,26,27,28]. In this type of system, face landmarks are extracted as the first step to identify eye and mouth features. Having the driver on a real-time camera would unavoidably introduce privacy concerns. In addition, the eyes typically occupy a fairly small portion of the images captured on camera, making the algorithms for detecting eye features vulnerable to head movements, fast-changing lighting conditions, and glares on glasses. These issues can be easily overcome by head-mounted devices, which offer a plug-and-play solution for existing cars that do not have cameras built in. In this line of research work, head-mounted eye-tracking glasses have long been used to examine driver drowsiness both in the lab and in natural driving scenarios [29]. Using SMI (SensoMotoric Instruments) eye-tracking glasses, Gao et al. [30] used a support vector machine (SVM) to analyze PERCLOS [31] to evaluate driving fatigue with time series data. Utilizing SMI eye-tracking glasses, Paletta et al. [32] obtained gaze density mapping by fusing scene camera images and gaze information to evaluate driver distraction. In addition to cameras, it is also possible to use other types of sensors to monitor eye state with glasses. For instance, He et al. [33] monitored the eye blink of drivers with the proximity sensors built into a Google glass. They examined how blink rates relate to driving dynamics, such as braking response time and lane keeping. These research works show that a wearable glass can be a practical solution for driver drowsiness detection.

## 3. Methods

**Figure 1:**
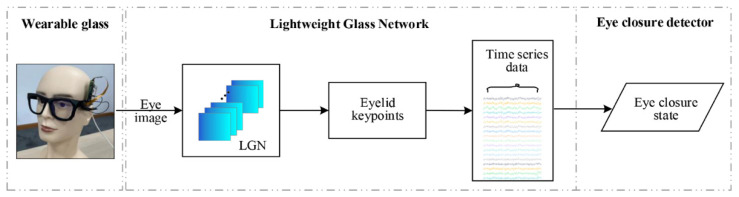
An eye closure monitoring system based on an in-house glass prototype and LGN.

As noted, drowsiness can be effectively monitored with the various visual features extracted from the eye, mouth, head pose, etc. [33]. This paper presents a drowsiness monitoring system based on a wearable glass prototype that monitors the eye closure state in real-time with a lightweight network. The three core modules of this system are illustrated in Figure 1. 

**Figure 2:**
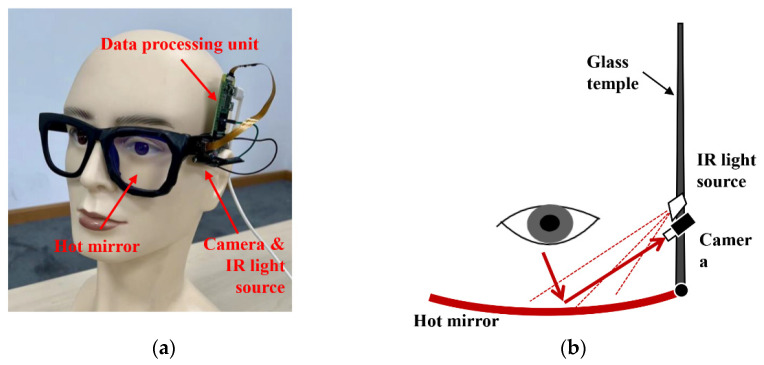
The hardware structure and the optical path of the wearable glass prototype. (**a**) Key components of the wearable glass; (**b**) The optical path of the camera.

**Wearable glass prototype:** At the core of this system is a wearable glass device that captures images of the eye area in real-time. The lens of the glass was replaced by a hot mirror to allow images of the eye to be captured by an RGB camera installed on the temple of the glass (see Figure 2). The RGB camera has an infrared filter, much like in a traditional video-based eye tracker, except that the camera is close to the eye and sits by instead of in front of the eye.

**Lightweight Glass Network:** The eye image is processed by a compact data processing unit and then forwarded to a lightweight deep learning network. For convenience, we will refer to this network as a Lightweight Glass Network, or LGN for short. For privacy considerations, the RGB camera was set to record low-resolution images, so it will not have a clear view of the iris. The LGN outputs 6 keypoints that mark the boundary of the eyelids, with which the eye area not covered by the eyelids is estimated and streamed as time series data.

**Eye closure detector:** Eye closure states, most notably blink, are monitored based on the size or the aspect ratio of the eye area.

The workflow of the proposed system is briefly summarized in Algorithm 1. As shown in Algorithm 1, real-time images obtained with the RGB camera and the hot mirror are first resized and normalized to meet the input requirements of the LGN. The LGN will output Gaussian probability maps (*E_G_*), and the eyelid keypoints (*E_k_*) are extracted based on the highest probability pixels of *E_G_*. Then, the open eye area (*S_k_*) is calculated and stored along with a time label (*S_kt_*). Finally, eye closure measures, e.g., eye blinks (*E_b_*), can be extracted from the open eye area sequence.
**Algorithm 1** The Workflow of Our Glass Prototype **Input:** Real-time eye images, *E_i_*;
**Output:** Eye closure measures, *E_b_*;1. **initial** *E_i_* = *Ø*, *E_b_* = *Ø*; 2. *E*_1_ ← Process the eye images to meet the input demand of LGN; 3. *E_G_* ← Obtain Gaussian probability maps with *E*_1_; 4. *E_k_* ← Extract eyelid keypoints from the highest probability pixels of *E_G_*; 5. *S_k_* ← Calculate eye open area with keypoints *E_k_*; 6. *S_kt_* ← (*S_k_, t*); 7. *E_b_* ← Extract eye closure measures from the open eye area sequence *S_kt_*; 8. **return** *E_b_*;

The structure of the glass prototype, the architecture of the LGN network, and the algorithm for eye closure detection are described in detail in the following subsections.

### 3.1. The Wearable Glass Prototype

The structure of the glass prototype and the working principle of a hot mirror is illustrated in Figure 2.

The glass consisted of a frame, a hot mirror that replaces the len, an infrared light source (LED), an RGB camera, and a data processing unit (Raspberry Pi Zero W). A hot mirror was used mainly because cameras installed on the rims (just below or in front of the eyes) unavoidably involve wires going through the hinges, which complicates the hardware design. This is one of the reasons that in commercial eye-tracking glasses, the temples cannot be folded like an ordinary pair of glasses. The other reason for using a hot mirror is that it helps to block infrared light in the environment from interfering with the eye images captured on the camera. However, the hot mirror allows the passage of visible light, so the glasses do not obstruct the view of the driver.

To maintain good contrast for the eye image reflected on the hot mirror, an infrared LED was installed by the camera to provide sufficient illumination in various lighting conditions (e.g., at night or under direct sunlight). Furthermore, the infrared light source can be used to control the quality of the image captured by the camera. If the infrared light is turned on, the camera will capture clear infrared images reflected on the hot mirror. Otherwise, the camera will lose track of the eye, as the captured image will be blurry and not suitable for eyelid keypoints detection. A Raspberry Pi Zero was chosen as the data processing unit because the I/O options allow for fast prototyping. This data processing unit can be replaced by any chip that has image processing and neural network capabilities.

### 3.2. The Lightweight Glass Network (LGN)

**Figure 3:**
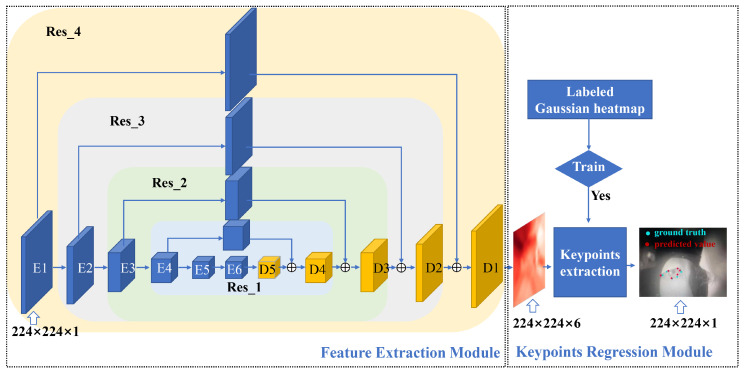
The architecture of the LGN network.

As shown in Figure 1, the eye image captured on camera is sent to a lightweight deep learning network (LGN) to extract the eyelid keypoints, and the architecture of the LGN is shown in Figure 3. As shown in Figure 3, the LGN has two modules: a Feature Extraction Module and a Keypoint Regression Module. Using a fourth-order network, the Feature Extraction Module extracts multi-scale features of the eye area; these features are sent to the Keypoints Regression Module to retrieve the pixel coordinates for the eyelid keypoints.

#### 3.2.1. Feature Extraction Module

As illustrated in Figure 2, the eye images are captured by a camera attached to the glass temple. Because the line of sight of the camera reaches the hot mirror with an angle, the captured eye images are inevitably distorted, making it difficult to estimate the eyelid keypoints. To resolve this issue, an hourglass network [34] was used in the Feature Extraction Module to extract features from eye images and subsequently fuse features with the raw eye images at different scales. The Feature Extraction Module will output 6 feature maps, each containing the position information of an eyelid keypoint.

The Feature Extraction Module shown in Figure 3 was adapted from an hourglass network, which was a fourth-order residual [35] module in essence. Nesting one single residual network into another will reduce the scale of a feature map by half. To obtain features at different scales and to estimate the keypoints from coarse to fine [36], 3 residual network modules (Res_1 ~ Res_3) were nested into the feature extraction module of LGN.

**Figure 4:**
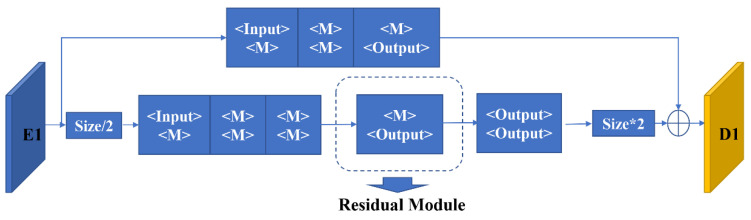
A single layer of the residual module in the Feature Extraction Module.

The structure of a single residual network is shown in Figure 4. A single residual network contains an upper branch and a parallel lower branch. When an image is processed by the three convolutional modules in the upper branch, the number of feature channels is changed based on the output settings, and meanwhile, the size of the input feature map remains unchanged. In the lower branch, the input feature map is down-sampled by the max-pooling layers, reducing the feature map by half. The down-sampled feature map is also sent to three convolutional modules, which share the same structure as the upper branch. For the output of the lower branch, an up-sampling process is performed via nearest neighbor interpolation to make the number of feature channels match its counterpart in the upper branch. In the last step, the feature maps output by the upper and lower branches are added in a pixel-wise fashion, and the fused features are used to compensate for gradient disappearance.

#### 3.2.2. Keypoints Regression Module

**Figure 5:**
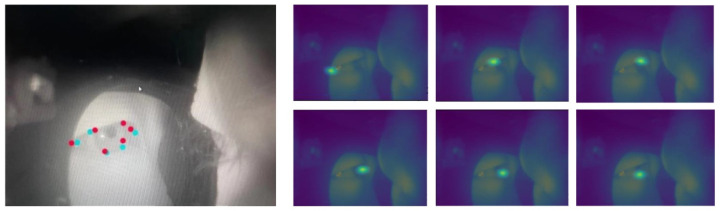
An illustration of the extracted eyelid keypoints (**left panel**) and the corresponding Gaussian probability maps estimated by the Feature Extraction Module (**right panel**).

The output of the Feature Extraction Module is a batch of 6 feature maps. Based on these feature maps, the Keypoints Regression Module will generate Gaussian probability maps for each of the eyelid keypoint through supervised training [37]. The eyelid keypoints were then estimated with the pixel position of the highest probability. An eye image with the extracted keypoints is illustrated in Figure 5 (left panel). The red dots are the model predicted keypoints, and the blue dots are the ground-truth labels. The Gaussian probability maps used to estimate the eyelid keypoints are presented in the right panel.

For model training, a Mean Squared Error (MSE) loss function was used to compare the predicted heatmap against a ground-truth heatmap, which consists of a 2D gaussian centered at an eyelid keypoint. The MSE-based loss function is presented in Equation (1), where *x_i_*(*h*, *w*) is the feature probability map, *i* represents the *i*-th predicted keypoint, $x_{i}^{*}\left(h,\;w\right)$ is the feature probability map of the *i*-th ground-truth keypoint. In Equation (1), *N* is the total number of eyelid keypoints, and *N* was 6 in the present work.

(1) $$Loss_{gaussian}=\frac{1}{N}\sum_{i}^{N}\sum_{hw}\|x_{i}\left(h,\;w\right)-x_{i}^{*}{\left(h,\;w\right)}^{2}\|$$

#### 3.2.3. Dataset and Training Setup 

**Figure 6:**
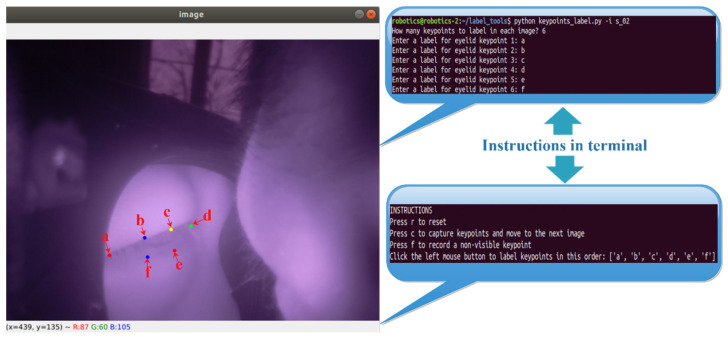
The labeling tools we used to annotate the image with eyelid keypoints.

As shown in Figure 2, the camera was attached to the glass temple and imaged the eye area from an extreme angle. This unique setup means that a new dataset must be first created to train the LGN network for eye closure estimation. The LGN network was intended to be a lightweight and easy-to-deploy network, so a small dataset is sufficient to ensure model convergence. Unfortunately, to the best of our knowledge, all freely accessible near-eye datasets cannot be used to train the LGN network, as the camera view of these open-source datasets is drastically different from that of our in-house glass system (see Figure 5 for a sample camera image). Therefore, we collected a total of 4400 images from 7 participants (4 males and 3 females) to create the small sample dataset. We then developed an OpenCV-based labeling tool to manually annotate the recorded eye images with six eyelid keypoints (see Figure 6). The labeled images and eyelid keypoints were used to construct Gaussian probability maps to improve model performance. During training, a subset of 1000 female and 1000 male eye images was randomly selected from the images for model evaluation.

Using this dataset, we trained the LGN network with an MSE-based loss function (Equation (1)). The training process involved minimizing this loss function over a set number of epochs. Raw eye images were preprocessed and sent to the LGN network, and the Gaussian probability maps constructed from the input images served as supervision for model training. A stochastic gradient descent (SGD) optimizer was used, and the initial learning rate was set to 0.001. The learning rate was reduced by a factor of 0.1 every 10 epochs during training. The training platform was a Dell Precision 3050 workstation equipped with an Nvidia GeForce RTX 2080 graphics card (Nvidia, Santa Clara, CA, USA), and the training process took about 1 h to complete.

## 4. Experiment

An experiment was carried out to validate our hardware prototype and the LGN network. As shown in Figure 3, the output of the lightweight network is a set of keypoints marking the boundary of the eyelids. With these keypoints, one can easily derive an eye aspect ratio or eye area measure, which can then be used to detect blinks or calculate a PERCLOS measure. Eye blink rate and PERCLOS are sensitive measures for drowsiness detection. The experiment presented here examined if the eye-closure measures (blink rate and PERCLOS) derived from the network output closely correlate with that from an industrially recognized EyeLink eye-tracker.

### 4.1. Testing Setup

**Figure 7:**
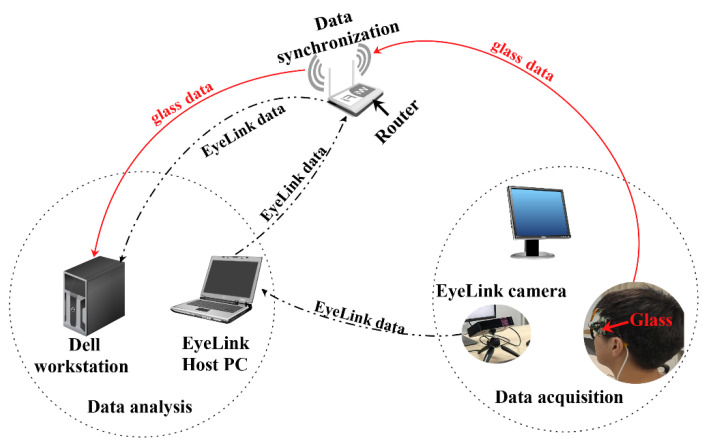
The testing setup used to validate the glass prototype and the LGN network.

The experimental setup is illustrated in Figure 7. In the experiment, the human participant wore the glass prototype and watched the visual contents on a 65-inch display. The glass prototype was powered by a portable phone charger and it wirelessly connected to a router to stream the captured eye images to a Dell Precision 3050 workstation. The workstation processed the images with the lightweight LGN network to retrieve eyelid keypoints and the eye area in real-time. An EyeLink eye-tracker was used to record pupil and gaze data simultaneously with the glass prototype. This video-based eye tracker has a tracking latency below 2 ms and the real-time eye tracking data, i.e., gaze position and pupil size, were accessible from the workstation via an Ethernet connection to the same router the glass prototype connected to.

The data recorded by the EyeLink eye-tracker contains information about the pupil area; however, it detects blinks only when the pupil is no longer visible to its infrared camera. So, the blinks reported by EyeLink are the period during which the eyes are fully closed. The blink durations reported by the tracker are much shorter than the actual durations of the eyelid movements. The hot mirror blocked infrared light and thus the EyeLink tracker could not see the left eye of the subject. In the experiment, the EyeLink tracker tracked the right eye only.

### 4.2. Testing Protocol

The goal of this experiment was to prove a concept. Three subjects (lab members) were recruited to participate in the experiment and each was tested for 15 min. At the beginning of a testing session, the EyeLink tracker was calibrated with a standard 9-point calibration procedure. Once recording started for both the EyeLink tracker and the glass prototype, the subject closed his/her eyes for about 15 s. This period of eye closure helps to align the glass and EyeLink data for offline analysis and comparison.

### 4.3. Measuring Eye Closure

Two eye closure measures were used to validate the glass prototype and the LGN output, notably, eye blink rate and PERCLOS. Eye blink rate was defined as the number of blinks per minute [38]. The field has seen several different definitions for PERCLOS [39,40], but it is frequently estimated as the percentage of eye closure per minute. Here, eye closure is defined as the duration during which the eyelids have covered over 80% of the eye area over a 1 min time window [40].

#### 4.3.1. Eye Closure Measures Based on EyeLink Pupil Data

Blinks are periods with missing samples with the EyeLink tracker. To estimate the blink rate, we used a 1 min moving window and counted the number of blinks detected by the EyeLink tracker. The tracker, however, does not provide an accurate estimate of the blink start and end time. So, a PERCLOS measure cannot be derived directly from the blink durations reported by the tracker. To obtain a better estimate of the start and end time of a blink, the present study reexamined the pupil area data with an intuitive yet robust algorithm [41,42]. During a blink, as the upper eyelid moves down, it does not occlude the pupil instantaneously, but rather, the tracker sees the pupil area quickly reduce to zero and rebounds as the eye reopens. The blink start and end time are the time points at which the EyeLink tracker starts and stops to report “no pupil”. However, the actual eyelid movement starts much earlier and stops much later than the “no pupil” period. The algorithm first searches backward in time to find a sample at which the pupil size no longer increases and marks it as the true start time, then searches forward in time to find a sample at which the pupil size no longer increases and marks it as the true end time. To calculate PERCLOS, we used 20% of the newly estimated blink duration as an approximation of the eye “closure” period of a blink.

#### 4.3.2. Eye Closure Measures Based on Glass Data

**Figure 8:**
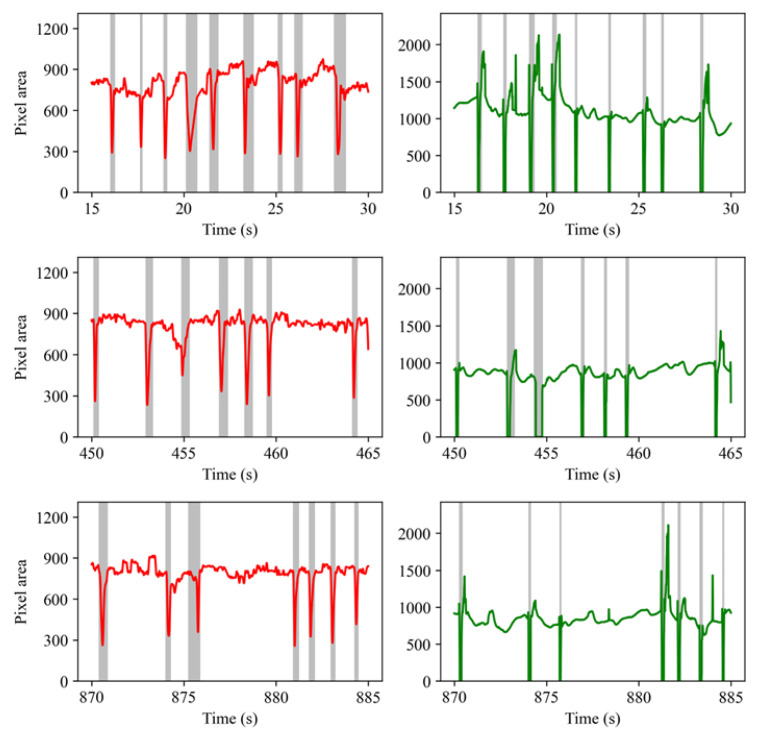
The eye blinks detected from the glass (**left panel**) and EyeLink (**right panel**) data recorded from Subject #2. Red lines represent the eye area data from our glasses; Green lines represent the pupil area data from the EyeLink eye-tracker. The detected blinks are marked with gray strips.

The LGN network output contains keypoints marking the boundary of the eyelids, with which the eye “area” can be easily estimated. With the eyelid keypoints, it is also possible to derive a height-width ratio (i.e., eye aspect ratio, or EAR for short) for drowsiness detection [43]. In the present study, an area measure was used for easy comparison with the pupil area data recorded by the EyeLink tracker. The LGN network outputs eye area at 30 Hz and these time series look much like EOG signals recorded with electrodes placed above and below the eye (see Figure 8 left panels). The signal drops as the upper eyelid moves down, reaches a local minimum as the eye is fully closed, and rebounds as the eyelid moves up. Thus, the present work borrowed an algorithm from a previous EOG study [44] to detect blinks. The working principle of this algorithm is similar to the one we used for blink detection on the EyeLink pupil size data. Briefly, the algorithm first up-samples the eye area signal to 250 Hz and then looks up the local minimums, then it searches the nearby samples for stable points at which the eye area signal no longer increases and marks these samples as blink start and end points. To estimate the blink rate, we used a 1 min moving window and counted the number of blinks detected by the algorithm. To estimate PERCLOS, we also used 20% of the estimated blink duration as an approximation of the eye “closure” period of a blink.

## 5. Results and Discussion

### 5.1. Performance of the Blink Detection Algorithms

**Figure 9:**
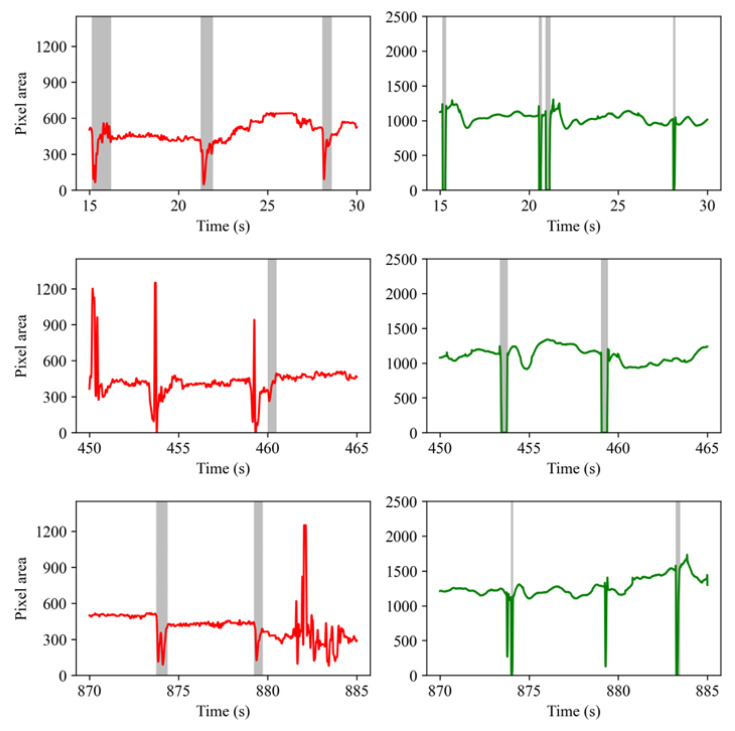
The eye blinks detected from the glass (**left panel**) and EyeLink (**right panel**) data recorded from Subject #3. Red lines represent the eye area data from our glasses; Green lines represent the pupil area data from the EyeLink eye-tracker. The detected blinks are marked with gray strips.

**Figure A1:**
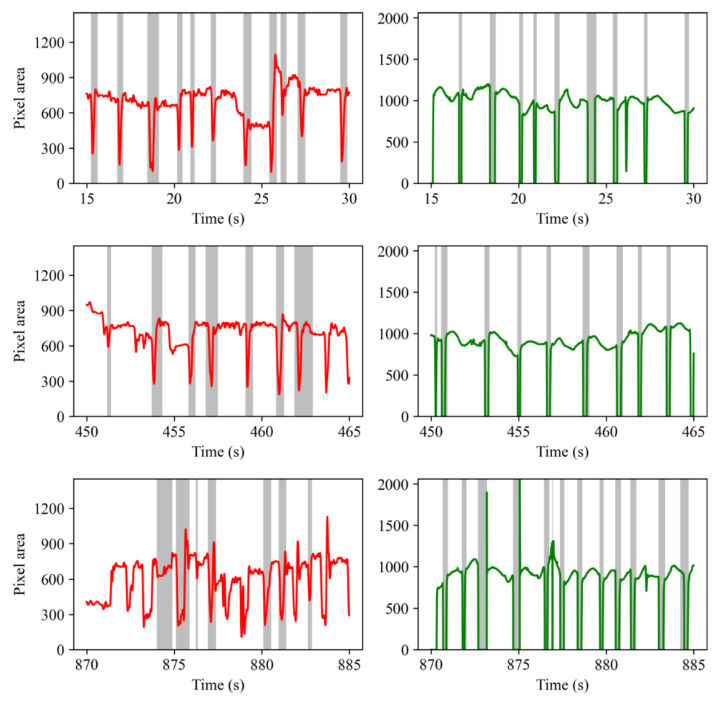
The eye blinks detected from the glass (**left panel**) and EyeLink (**right panel**) data recorded from Subject #1. Red lines represent the eye area data from our glasses; Green lines represent the pupil area data from the EyeLink eye-tracker. The detected blinks are marked with gray strips.

Two slightly different algorithms were used to estimate the start and end time of blinks for the EyeLink pupil area data and glass eye area data. Figure 8 shows the blinks detected from the glass eye area data (left panel) and the EyeLink pupil area data (right panel) recorded from Subject #2 (see Figure 9 and Figure A1 for example blinks detected from the data recorded from Subject #3 and #1). The three rows contain data recorded at the start of, and 7.5 and 14.5 min into a recording session. The vertical strips in Figure 8 represent the eye blinks. As is clear from this figure, the blinks detected from the glass output largely agree with those detected from the EyeLink pupil data.

It is clear that the blinks detected from the glass data were much longer than that detected from the Eyelink pupil area data. This was no surprise as the glass prototype measured the eye area and the EyeLink tracker measured the pupil area only. When the upper eyelid moves down, the camera will lose sight of the pupil before the eyelids reach the bottom; when the eyelid moves up, the pupil will likely become fully visible before the eyelid movement stops. This between-device difference will not affect the blink rate measure, but the PERCLOS measure may differ across devices. The most important message in the figure is that the glass prototype and the LGN network output can be used to correctly detect eye blinks.

The total number of blinks detected from the glass and EyeLink data and the mean blink durations are presented in Table 1. Overall, the glass prototype detected on average 93.9% of the eye blinks reported by the Eyelink trackers. However, bear in mind that there were false blink detections by both the EyeLink tracker and the glass prototype. For instance, the EyeLink tracker detects blinks based on missing data samples; however, missing samples could also be the result of device failure or the head moving outside the trackable range. Some blink detection failures are illustrated in Figure 9. In the first row, two temporally adjacent blinks were detected from the EyeLink data between 20–22 s; however, only one blink was detected from the glass eye area data. The failure on the glass side was most likely because the sampling rate of the glass prototype was much lower (30 Hz) and consequently, the glass is not sensitive to very short blinks. However, this is not always the case. Note that in Figure 9 (third row, left panel) the glass also recorded two short and consecutive signal troughs that were seen in the EyeLink pupil signal, showing that the LGN network was very sensitive to eye closures.

### 5.2. Blink Rate and PERCLOS

**Figure 10:**
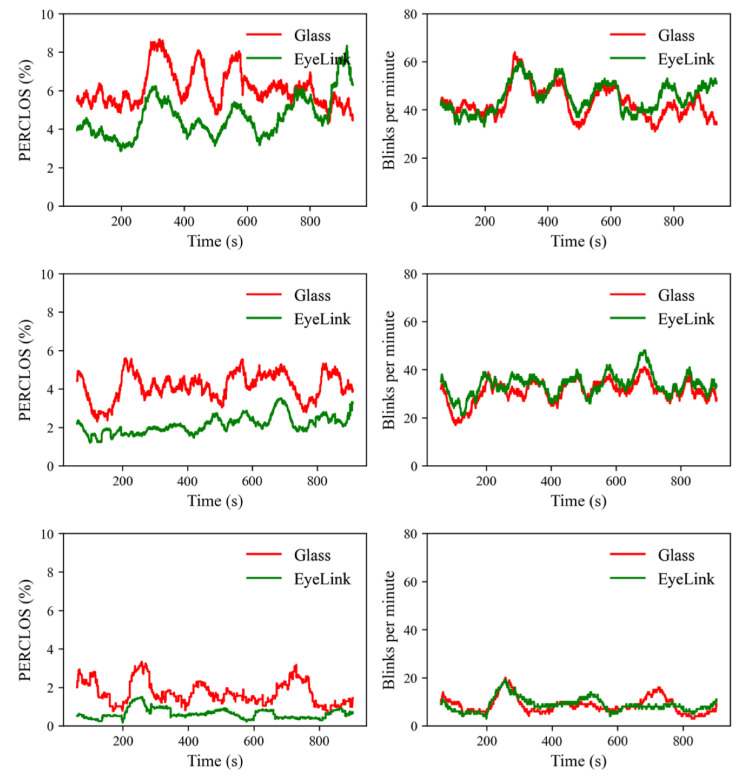
The PERCLOS (**left panel**) and blink rate (**right panel**) measures calculated from the three recording sessions.

To validate the PERCLOS and blink rate measures derived from the output of the LGN network, a 1 min moving window was used to calculate the PERCLOS and blink rate on the time series data, with steps of 0.1 s. The PERCLOS (left panel) and blink rate (right panel) over the three recording sessions are presented in Figure 10.

There is no uniformly agreed definition of PERCLOS. Nevertheless, most people define eye “closure” as the eyelids covering over 80% of the pupil or eye area. As a close approximation, the present study defines eye closure as 20% of the duration of an eye blink and PERCLOS is the percentage of eye closure time over a 1 min interval. 

As shown in Figure 10, the PERCLOS measures derived from the two devices co-varied in some time windows. Several methods have been developed to quantify the similarity or distance between time series [45]. We chose the Pearson correlation to quantify the temporal similarity in PERCLOS between the two devices because a correlation coefficient is straightforward to interpret. Specifically, a correlation coefficient of 0 and 1.0 would suggest no similarity and perfect match between two time series, respectively. In this analysis, we first calculated the correlation in PERCLOS between the two devices for each subject, then we tested the correlation coefficients against 0 with a one-sample *t*-test. A correlation coefficient (*r*) of 0.10, 0.30, and 0.50 is regarded as small, medium, and large, respectively [46]. The results revealed medium-sized correlations between the two devices (*r_mean_* = 0.286, *r_sd_* = 0.118), which were stronger than 0, *t*(2) = 4.194, *p* = 0.052. The moderate correlation between the two devices is understandable as eye closure was defined differently across the two devices. The pupil makes up only a small portion of the eye area. For the glass prototype, eye closure measures the period during which the eyelids covered the eyeball; for the EyeLink tracker, eye closure was the period during which the eyelids covered the pupil.

As is clear from Figure 10, the blink rate estimated from the glass output largely matches that estimated from the EyeLink tracker. Statistical analysis revealed strong correlations in blink rate between the two devices (*r_mean_* = 0.729, *r_sd_* = 0.110), which were stronger than 0 as well, *t*(2) = 11.511, *p* = 0.007. As discussed in the previous section, the total number of blinks identified from the glass output on average was about 93.9% of that identified by the EyeLink tracker. This performance in detecting blinks was outstanding considering that the output speed of the glass (30 Hz) was approximately 1/17 of the speed of the EyeLink tracker (500 Hz).

These results clearly show that the glass prototype was a sensitive measurement device for eye closures. The effectiveness of the PERCLOS and blink rate measures in drowsiness detection should be further validated with empirical studies that manipulate the level of drowsiness of vehicle drivers. Nevertheless, the present results clearly show that the glass prototype and the LGN network provide a viable solution for deriving measures that are sensitive to driver drowsiness.

## 6. Conclusions

This study focuses on a key issue in traffic safety, i.e., developing a wearable, reliable, and user-acceptable drowsiness detection system. Remote camera-based systems rely on in-cabin camera(s) that monitor the facial and body features of the driver; privacy concerns among the drivers and the required hardware modifications may seriously hinder market adoption. 

Compared to classic remote camera-based systems, the proposed wearable glasses utilize close-up images of the eyes instead of relying on the facial and body features of the driver. This approach allows for precise quantification of eye closure using sensitive measures such as PERCLOS and blink rate, effectively circumventing privacy issues associated with traditional methods. 

The glass prototype developed in the present work images the eye area via a hot mirror and measures eye closure with a lightweight deep learning network (LGN). The LGN network extracts keypoints that mark the eye area between the eyelids. The experimental results showed that the wearable glass prototype, with the LGN network in its core, was highly effective in detecting eye blinks; it detected on average 93.9% of the eye blinks reported by a gold standard EyeLink eye-tracker. More importantly, the blink rate derived from the glass output was highly consistent with the EyeLink tracker, showing that the LGN framework is a computationally efficient yet viable solution for real-world applications.

To conclude, the wearable glass presented in this paper is an efficient and reliable solution for driver eye closure monitoring. In future work, the Raspberry Pi Zero board can be replaced with a small factor chip to improve power efficiency. Moreover, efforts will be made to make the glass lighter and more comfortable to wear. An experiment would also be conducted to validate the drowsiness detection performance of our glass in naturalistic driving scenarios.

## Figures and Tables

**Table 1:** Total number of blinks detected from the glass and EyeLink data and the average blink duration.

	Blink Count		Blink Duration *	
Subject	Glass	Eyelink	Glass	Eyelink
s1	666	706	0.43(0.20)	0.30 (0.17)
s2	472	514	0.40 (0.21)	0.19 (0.12)
s3	131	137	0.59 (0.31)	0.21 (0.14)

* Blinks longer than 2.0 s (11 in total) were excluded from the table; numbers in the parentheses are SDs.

## Data Availability

The data that support the findings of this study are available from the corresponding author, S.Z, upon reasonable request.

## References

[1] Sheldon Z., Gary R.V. (2014). APA Dictionary of Statistics and Research Methods.

[2] Patte D. (2019). Cambridge Dictionary.

[3] Addanki S.C., Jaswanth N., Assfalg R. (2020). Analysis of traffic related factors and vehicle environment in monitoring driver’s driveability. Int. J. Intell. Transp. Syst. Res..

[4] Wise J.M., Heaton K., Patrician P. (2019). Fatigue in long-haul truck drivers: A concept analysis. Workplace Health Saf..

[5] Williamson A., Lombardi D.A., Folkard S., Stutts J., Courtney T.K., Connor J.L. (2011). The link between fatigue and safety. Accid. Anal. Prev..

[6] Tefft B.C. (2012). Prevalence of motor vehicle crashes involving drowsy drivers, United States, 1999–2008. Accid. Anal. Prev..

[7] Owens J.M., Dingus T.A., Guo F., Fang Y., Perez M., McClafferty J., Tefft B.C. (2018). Prevalence of Drowsy-Driving Crashes: Estimates from a Large-Scale Naturalistic Driving Study.

[8] Cyganek B., Gruszczyński S. (2014). Hybrid computer vision system for drivers′ eye recognition and fatigue monitoring. Neurocomputing.

[9] Bafna T., Hansen J.P. (2021). Mental fatigue measurement using eye metrics: A systematic literature review. Psychophysiology.

[10] Bitkina O.V., Park J., Kim H.K. (2021). The ability of eye-tracking metrics to classify and predict the perceived driving workload. Int. J. Ind. Ergon..

[11] Fu X., Guan X., Peli E., Liu H., Luo G. (2012). Automatic calibration method for driver′s head orientation in natural driving environment. IEEE Trans. Intell. Transp. Syst..

[12] Dziuda Ł., Baran P., Zieliński P., Murawski K., Dziwosz M., Krej M., Piotrowski M., Stablewski R., Wojdas A., Strus W. (2021). Evaluation of a fatigue detector using eye closure-associated indicators acquired from truck drivers in a simulator study. Sensors.

[13] Hari C.V., Sankaran P. (2021). Driver distraction analysis using face pose cues. Expert Syst. Appl..

[14] Hedges & Company (2021). Research on Vehicle-Based Driver Status/Performance Monitoring; Development, Validation, and Refinement of Algorithms for Detection of Driver Drowsiness.

[15] Venkata Phanikrishna B., Jaya Prakash A., Suchismitha C. (2021). Deep review of machine learning techniques on detection of drowsiness using EEG signal. IETE J. Res..

[16] Ramzan M., Khan H.U., Awan S.M., Ismail A., Ilyas M., Mahmood A. (2019). A survey on state-of-the-art drowsiness detection techniques. IEEE Access.

[17] Young L.R., Sheena D. (1975). Survey of eye movement recording methods. Behav. Res. Methods Instrum..

[18] Tian Y., Cao J. (2021). Fatigue driving detection based on electrooculography: A review. EURASIP J. Image Video Process..

[19] Xue Q.H., Zheng W.L., Lu B.L. Driving fatigue detection with fusion of EEG and forehead EOG. Proceedings of the 2016 International Joint Conference on Neural Networks (IJCNN).

[20] Schmidt J., Laarousi R., Stolzmann W., Karrer-Gauß K. (2018). Eye blink detection for different driver states in conditionally automated driving and manual driving using EOG and a driver camera. Behav. Res. Methods.

[21] Pandey N.N., Muppalaneni N.B. (2022). A survey on visual and non-visual features in Driver’s drowsiness detection. Multimed. Tools Appl..

[22] Maior C.B.S., das Chagas Moura M.J., Santana J.M.M., Lins I.D. (2020). Real-time classification for autonomous drowsiness detection using eye aspect ratio. Expert Syst. Appl..

[23] Cheng Q., Wang W., Jiang X., Hou S., Qin Y. (2019). Assessment of driver mental fatigue using facial landmarks. IEEE Access.

[24] Bamidele A.A., Kamardin K., Abd Aziz N.S.N., Sam S.M., Ahmed I.S., Azizan A., Bani N.A., Kaidi H.M. (2019). Non-intrusive driver drowsiness detection based on face and eye tracking. Int. J. Adv. Comput. Sci. Appl..

[25] Madireddy R., Anudeep D.S.K., Poorna S.S., Anuraj K., Krishna M.G., Balaji A., Venkat D.J. (2021). Driver Drowsiness Detection System Using Conventional Machine Learning. Computer Networks and Inventive Communication Technologies: Proceedings of Third ICCNCT 2020.

[26] Kumar U.M., Singh D., Jugran S., Punia P., Negi V. (2018). A system on intelligent driver drowsiness detection method. Int. J. Eng. Technol..

[27] Deng W., Wu R. (2019). Real-time driver-drowsiness detection system using facial features. IEEE Access.

[28] Liu Z., Peng Y., Hu W. (2020). Driver fatigue detection based on deeply-learned facial expression representation. J. Vis. Commun. Image Represent..

[29] Schweizer T., Wyss T., Gilgen-Ammann R. (2022). Detecting Soldiers’ Fatigue Using Eye-Tracking Glasses: Practical Field Applications and Research Opportunities. Mil. Med..

[30] Gao X.Y., Zhang Y.F., Zheng W.L., Lu B.-L. (2015). Evaluating driving fatigue detection algorithms using eye tracking glasses. Proceedings of the 2015 7th International IEEE/EMBS Conference on Neural Engineering (NER).

[31] Dinges D.F., Grace R. (1998). PERCLOS: A Valid Psychophysiological Measure of Alertness as Assessed by Psychomotor Vigilance.

[32] Paletta L., Schwarz M., Wollendorfer C., Perko R. (2021). Information Fusion for Driver Distraction Studies Using Eye Tracking Glasses. Advances in Human Aspects of Transportation: Part I.

[33] He J., Choi W., Yang Y., Lu J., Wu X., Peng K. (2017). Detection of driver drowsiness using wearable devices: A feasibility study of the proximity sensor. Appl. Ergon..

[34] Yang J., Liu Q., Zhang K. Stacked hourglass network for robust facial landmark localization. Proceedings of the IEEE Conference on Computer Vision and Pattern Recognition Workshops.

[35] He K., Zhang X., Ren S., Sun J. Deep residual learning for image recognition. Proceedings of the IEEE Conference on Computer Vision and Pattern Recognition.

[36] Newell A., Huang Z., Deng J. (2017). Associative embedding: End-to-end learning for joint detection and grouping. Advances in Neural Information Processing Systems.

[37] Cao Z., Simon T., Wei S.E., Sheikh Y. Realtime multi-person 2D pose estimation using part affinity fields. Proceedings of the IEEE Conference on Computer Vision and Pattern Recognition.

[38] David F.D., Malissa M.M., Greg M., Powell J.W. (1998). Evaluation of Techniques for Ocular Measurement as an Index of Fatigue and as the Basis for Alertness Managemen.

[39] Sommer D., Golz M. (2010). Evaluation of PERCLOS based current fatigue monitoring technologies. Proceedings of the 2010 Annual International Conference of the IEEE Engineering in Medicine and Biology.

[40] Qing W., BingXi S., Bin X., Zhao J. (2010). A perclos-based driver fatigue recognition application for smart vehicle space. Proceedings of the 2010 Third International Symposium on Information Processing.

[41] Ghose U., Srinivasan A.A., Boyce W.P., Chng E.S. (2020). PyTrack: An end-to-end analysis toolkit for eye tracking. Behav. Res. Methods.

[42] Hershman R., Henik A., Cohen N. (2018). A novel blink detection method based on pupillometry noise. Behav. Res. Methods.

[43] Dari S., Epple N., Protschky V. (2020). Unsupervised blink detection and driver drowsiness metrics on naturalistic driving data. Proceedings of the 2020 IEEE 23rd International Conference on Intelligent Transportation Systems (ITSC).

[44] Agarwal M., Sivakumar R. (2019). Blink: A fully automated unsupervised algorithm for eye-blink detection in eeg signals. Proceedings of the 2019 57th Annual Allerton Conference on Communication, Control, and Computing (Allerton).

[45] Liao T.W. (2005). Clustering of time series data—A survey. Pattern Recognit..

[46] Cohen J. (1992). Quantitative methods in psychology: A power primer. Psychol. Bull..

